# The integration of the treatment for common mental disorders in primary care: experiences of health care providers in the MANAS trial in Goa, India

**DOI:** 10.1186/1752-4458-5-26

**Published:** 2011-10-03

**Authors:** Bernadette Pereira, Gracy Andrew, Sulochana Pednekar, Betty R Kirkwood, Vikram Patel

**Affiliations:** 1 Sangath, Alto-Porvorim, Goa 403521, India; 2 Faculty of Epidemiology & International Health, London School of Hygiene & Tropical Medicine, Keppel Street, London, WC1E 7HT, UK

## Abstract

**Background:**

The MANAS trial reported that a Lay Health Counsellor (LHC) led collaborative stepped care intervention (the "MANAS intervention") for Common Mental Disorders (CMD) was effective in public sector primary care clinics but private sector General Practitioners (GPs) did as well with or without the additional counsellor. This paper aims to describe the experiences of integrating the MANAS intervention in primary care.

**Methods:**

Qualitative semi-structured interviews with key members (n = 119) of the primary health care teams upon completion of the trial and additional interviews with control arm GPs upon completion of the outcome analyses which revealed non-inferiority of this arm.

**Results:**

Several components of the MANAS intervention were reported to have been critically important for facilitating integration, notably: screening and the categorization of the severity of CMD; provision of psychosocial treatments and adherence management; and the support of the visiting psychiatrist. Non-adherence was common, often because symptoms had been controlled or because of doubt that health care interventions could address one's 'life difficulties'. Interpersonal therapy was intended to be provided face to face by the LHC; however it could not be delivered for most eligible patients due to the cost implications related to travel to the clinic and the time lost from work. The LHCs had particular difficulty in working with patients with extreme social difficulties or alcohol related problems, and elderly patients, as the intervention seemed unable to address their specific needs. The control arm GPs adopted practices similar to the principles of the MANAS intervention; GPs routinely diagnosed CMD and provided psychoeducation, advice on life style changes and problem solving, prescribed antidepressants, and referred to specialists as appropriate.

**Conclusion:**

The key factors which enhance the acceptability and integration of a LHC in primary care are training, systematic steps to build trust, the passage of time, the observable impacts on patient outcomes, and supervision by a visiting psychiatrist. Several practices by the control arm GPs approximated those of the LHC which may partly explain our findings that they were as effective as the MANAS intervention arm GPs in enabling recovery.

## Introduction

Mental health is central to the values and principles of the Alma Ata Declaration, adopted by nations from around the world at the landmark International Conference on Primary Health Care (1978). It is widely acknowledged that integrating mental health care into primary care is the only viable way of closing the treatment gap for Common Mental Disorders (CMD) [1]. There are several reasons for this position, including the large burden of mental disorders in primary health care settings, the lack of specialist human resources and the stigma associated with mental health care [1]. However, currently the recognition and treatment of CMD among the large number of people who attend primary care settings with CMD is extremely inadequate [2] with a consequent large unmet need for care [3]. Despite the substantial evidence base on the efficacy of pharmacological and psychosocial treatments delivered by non-specialist health workers in primary care settings [4,5], efforts to integrate these into routine primary health care have faced numerous challenges. One major challenge is the lack of human resources with the relevant skills to deliver mental health care. Competing priorities often lead to existing human resources being unavailable or unmotivated to take responsibility for this task.

The MANAS intervention sought to overcome some of these challenges through the use of trained lay counsellors working in a collaborative care framework with the primary care physician and a visiting mental health specialist. The intervention was evaluated through a cluster randomized controlled trial in public and private primary health care settings. Altogether, 2796 patients with a CMD from 24 primary health care clusters participated with over 80% follow up rates at 2, 6 and 12 month, making this the largest trial in psychiatry from the developing world [6]. The primary findings of the trial revealed that the intervention led to significant improvements in recovery rates from CMD in the public sector clinics. There was no impact, however, in the private sector where the 'control' arm General Practitioners (GPs) were able to achieve outcomes comparable to those in the Manas intervention arm [6]. We hypothesize that this could be due to the similarity of the 'usual care' adopted by these GPs to the principles of the MANAS intervention. This paper describes the experiences of integrating the MANAS model into primary care. It also explores the care provided by the control arm GPs to 'unpack' the findings suggesting non-superiority of the collaborative care model in that sector.

## Method

### Setting

The MANAS trial was conducted in Goa, a state in west India with a population of 1.4 million. It was implemented by Sangath, a Non Governmental Organization, in collaboration with the London School of Hygiene and Tropical Medicine, the Government of Goa's Directorate of Health Services, the Voluntary Health Association of Goa and private general medical practitioners (GPs). The MANAS trial was carried out in two consecutive phases, first in 12 government run primary health care facilities (Primary Health Centres or PHC's) and then in 12 private GP practices. PHCs typically comprised an average of 2-3 doctors and a team of non-medical workers (nurses, community health workers, and administrative staff). Their practice style is best described as a 'collective clinic centred model' where patients have little privacy during consultations and may see different doctors on different visits. All services, including medications available in the PHC pharmacy, are free. In contrast, GPs are single practitioner operated private enterprises where all services are on a cash-basis and all prescribed medications must be purchased. Their practices are small and have few ancillary staff beyond a secretary. However, the patient typically will see the same doctor on each visit and consultations take place in a private room with a closed door; this model of care has been referred to as an 'individual, personalized client-centred care model'[7].

### Overview of Trial Design

The study was a cluster randomized trial conducted in two consecutive phases, first with PHCs and then with GPs, randomised to deliver either the MANAS Collaborative Stepped Care intervention or Enhanced Usual Care control. Patients in all facilities were screened for CMD by a trained lay person who was called the Health assistant (HA) using the 12 item General Health Questionnaire (GHQ-12) with a cut-off score of 5/6; the results of the screening were provided to the primary care physician. The MANAS intervention involved three key members: the Lay Health Counsellor (LHC), the primary care physician and a visiting psychiatrist ("Clinical Specialist"). Full details of the development and content of the intervention have been previously published [8]. The LHC was a female college graduate, recruited from the local community, who was trained to lead the intervention in a structured two month course to deliver a range of psychosocial treatments (psychoeducation, interpersonal therapy, referral to appropriate agencies, adherence support) for CMD. She acted as a case manager for all patients who screened positive for CMD and took overall responsibility for the intervention delivery, working in close collaboration with the primary care physician and the Clinical Specialist for all non-drug treatments and supporting adherence with antidepressants for those who were prescribed the drug by the primary care physician. In the control facilities, the results of screening were provided to the physicians by the Health Assistant along with the treatment guidelines manual; no other intervention was offered.

### Interview participants and recruitment

All key members of the primary health care teams in the MANAS intervention and control clinics (Primary Care Doctors/GPs, Lay Health Counsellors, the Clinical Specialists and ancillary staff of the clinics) were invited to participate upon completion of the trial. In addition, all the six private practitioners in the control arm were invited for an additional interview upon completion of the outcome analyses of the MANAS trial which revealed non-inferiority of this arm. Written informed consent was a pre-requisite for participation.

### Data collection and analysis

The interview guides for the semi- structured interviews used in this study were developed, piloted and refined to elicit information on the experiences of the respondents during the trial. The first part of the guide covered the role of the respondent in intervention delivery and his or her description of the role played by the other key persons delivering the various components of the intervention. The second part of the guide explored experiences with each component of the intervention and the respondent's opinion on the program as a whole. The control arm participants were only asked about the screening component (i.e. detection of CMD) as this was the only component added to the usual care in those clinics. The secondary data collected from the six GPs in the control arm sought to elicit information about their practices in management of patients with CMD with the goal of comparing these with the MANAS intervention components.

All interviews were conducted by the first two authors (GA, BP) who were not directly involved in the intervention, within two months of completing the trial, in their respective setting, maintaining privacy and confidentiality. All interviews were audio-recorded. The audio-recorded interviews were transcribed verbatim and all local language interviews were translated into English. All interviews were entered into AtlasTi (version 4.2) software, and coded in an iterative manner using a thematic framework of analyses. The data was categorized into the following broad themes: the experiences of integrating the intervention in the primary care setup, the roles played by the various actors, challenges faced, and the strategies used to overcome the challenges. The data for the six GP's in the control arm were coded in a similar manner and mainly focused on how they managed patients with CMD before and during the MANAS program and views about why they achieved comparable results.

### Ethical issues

#### 

All respondents were assured that participation was voluntary and that no information identifying the individuals would appear in the reports. Written consent was obtained from participants that included consent for audio-recording. Approval was obtained from the IRBs of Sangath and the LSHTM.

## Results

A total of 119 subjects participated in this study (Table 1) of whom 48 were from the control arm clinics. Findings are presented in two sections; the first addresses the experiences of the integration of the MANAS program in PHCs and GPs; the second addresses the practices of the control arm GPs.

**Table 1:** Participants in MANAS qualitative study

Role in the MANAS program	N
PHC doctors and GPs	31
Health Counsellor	17
Health Assistant (responsible for screening)	28
Clinical Specialist (visiting psychiatrist)	2
Ancillary Staff (Registration Clerk, Pharmacists, Staff Nurses, Auxiliary Nurse Midwife, Extension Officer)	41
TOTAL	119

### Experiences of integration

#### Screening for CMD

The method for detection of CMD was through screening with a validated questionnaire by a Health Assistant (HA), recruited for this task. The benefits of the screening were appreciated by most respondents who reported that screening provided an opportunity for patients to talk about their health and their problems. A key element for the acceptability of the screening was the polite, cooperative and friendly nature of the HA and that the screening involved asking about personal concerns which were not usually covered during health care consultations.

"She (HA) had a nice way with the patients. She made them feel comfortable, she then inquired about their health complaints. People liked that way." [PHC clerk]

"Screening makes the patients feel better. Nobody asks those questions, how you are feeling, how you are getting along in life etc. It also makes the patients think because such questions are never asked by anybody - it is for them to look at whether they have got palpitations or any other problem. Screening made all the patients to think about their problem." [PHC doctor]

While just over half the PHC doctors (12/20) and most GPs (8/11) reported that they were diagnosing CMDs prior to the program, almost all emphasized that the screening and further categorization of CMD as mild or moderate/severe and recording on a patient card helped them in sharpening their diagnostic abilities and providing treatment.

"The score card was very helpful in coming to the clear cut diagnosis. Before the program we were not tapping much of depressed cases and we would label them as psychosomatic and most of the times give them vitamins and tranquilizers, if severe cases then we would refer them to the psychiatrist. Now we prescribe anti depressants to these patients or send them for counseling." [PHC doctor]

"Screening helped me; it reinforced my ideas and removed certain misconceptions from my mind like certain false cases that I would have treated for depression." [GP]

Several challenges to screening were reported, the most common being the hesitance of patients to participate in the screening since they were in a hurry and feared that they might miss their turn to see the doctor. To ease these fears, the clinic staff provided information and reassured patients about the benefits of the screening in terms of its value for the overall medical assessment by the doctor. Doctors also referred unscreened patients without the patient card back for screening. Health Assistants adopted a number of strategies, reassuring patients that they would accompany them to the doctor in the event that they missed their place in the queue.

"Patients are always in a hurry, most challenging was to ask for their time from their busy schedule. They would feel that they would miss the doctor so I had to convince them. Patients never said that they will not give time. All would depend on how the introduction is given to the patient. Once patients are convinced then other procedures are very easy." [HA]

*"In the beginning the patients used to ask why they are calling us. We used to tell them that she (HA) would ask you some questions on health and you have to answer them. She (HA) has patience and would talk very nicely with the patient." *[GP nurse]

Other challenges included engaging with patients who had been non-cases during a previous screening encounter and who hesitated for re-screening; patients were re- screened if they re-attended the practice only if there was at least one month since the previous screening. Some patients expected monetary benefits and some patients influenced others not to go for screening. Some HAs reported difficulties in handling too many patients for screening and the rude behaviour of a few male patients. These challenges were handled by providing explanation about the benefits of the program, in particular its impact on the treatment the patient could receive that day, and allocating an additional HA for very busy clinics. The HA's also attributed their skills to the training on how to handle difficult situations and the accompanying role plays. They observed that over time when the doctors received positive feedback from patients about the treatment they got more involved in the program.

*"Gradually doctors became aware of the program and they began to take interest in the program like referring unscreened patients. They also began to refer patients for screening from the hospital ward, PHC sub-centers and other people known to them. Patients would tell the doctors that they had felt better, tablets had helped them. Based on the feedback of the patients, doctor's views about the program changed and they cooperated." *[HA]

#### Medication practice by primary care doctors

The majority of the PHC doctors observed a change in their prescription behaviour, notably reduced prescription of benzodiazepines (15/20) and vitamins and tonics (13/20) and increased prescription of antidepressants.

*"Fluoxetine was not supplied by the DHS *(Directorate of Health Services) *so I could not do anything. I had to prescribe diazepam or B complex. When MANAS program made it (*Fluoxetine*) available then we had an upper hand to prescribe it to our patients." [PHC doctor]*

Similar changes were observed in GP prescription of benzodiazepines, but most continued their practice of prescribing vitamins and tonics.

*"It is a tradition in my area even before I started my practice. Psychologically patients feel that they should have one bottle of tonic or some vitamins. Many a times they ask doctor "we want one tonic bottle". Sometimes vitamins are really required especially anemic patients." *[GP]

When doctors were asked about the experiences of patients who received Anti-depressants (ADT), less than half of the doctors pointed out that patients were complaining of side effects like nausea, giddiness, dryness of mouth and gastritis. The doctors handled side effects by discontinuing ADT, switching to another ADT, or reassuring the patient to continue the medication in the expectation that tolerance would develop. The majority of doctors in both sectors expressed much greater confidence in prescribing ADT to patients with CMD as a result of the program.

"Now I am comfortable prescribing antidepressants to patients because I have gained confidence in this drug. There have been many patients, about seven to eight of them who have been prescribed antidepressants. I felt that so many patients have shown positive results, why I can't continue with the same medication." [PHC doctor]

#### Psychosocial interventions delivered by the Lay Health Counsellor

The role of the LHC was to be a case manager and provide psychoeducation to all patients with CMD, support adherence for those receiving ADT, and provide interpersonal therapy for patients with moderate/severe depression or those who did not respond to ADT. Intervention materials used by the LHCs included handouts and a flip chart for psychoeducation. More than half of the LHC's reported that the patient card with the screening results aided them in planning the intervention for the patient. Almost all LHC's observed that handouts helped patients to remember the information provided by them, and created awareness among the family members as less literate patients were instructed to get it read by their family members, thus facilitating family support. The flip chart was observed to facilitate the psychoeducation by helping the patients understand the content better and acting as a guide to the LHC.

Most of the clinic staff mentioned that LHCs were accepted by the patients as they were polite, friendly, spent time building rapport with the patients and respected confidentiality. However, the LHCs described a number of challenges that they faced in the delivery of the psychological treatments. During the early days of the program, some LHCs reported difficulty remembering the guidelines of psychoeducation, forgetting, for example, to provide advice on some symptoms or inquire about substance abuse. Some also experienced difficulty in handling talkative patients, working with patients with extreme social difficulties, counseling people with alcohol related problems, and communicating with elderly patients who found it difficult to follow the instructions. Less common difficulties were resentment by patients when the LHCs inquired about suicidal ideas and, particularly in the GP sector, patients hesitating to open up as they were known to the doctors and the staff. Some patients requested financial assistance and some refused treatment as they did not feel it would help them.

*"Some (patients faced) social difficulties like financial problem which is mainly due to seasonal work, daily wages, and alcoholism. Another problem was patients not having proper documentation to apply for social schemes e.g. unregistered marriage, so a woman cannot apply for widow pension. So I found that there is no proper channel to improve their social difficulties and their problem remains same and this worsens their health condition. But I tried to give them information about various available schemes and how to follow the procedure and some even applied for it." *[LHC]

The majority of LHCs reported that experience in the clinics, training before the program and monthly peer group supervision during the program comprising, for example, role plays of difficult scenarios, gave them confidence to overcome the challenges they faced. With experience, the LHCs adopted a flexible approach of delivering psycho-education, improving their skills in asking about suicidal ideas, and providing concrete information on welfare schemes and referral agencies to address social difficulties and reviewing progress on these issues in follow up appointments. The LHCs observed that providing explanations about the importance of treatment and explaining the mind-body link also helped engage patients who were skeptical about the program's effectiveness. LHCs also learnt to emphasize confidentiality which helped patients become more comfortable in talking to them.

*"I stressed to every patient that whatever they would confide in me would remain confidential. I felt these words would make the patient feel more comfortable with me and they would ventilate their feelings more easily. Another aspect of psychoeducation which I felt has helped a lot was the mind-body link which included the patient explaining the stressors in their life which in turn would affect their health*." [LHC]

LHCs delivered Interpersonal Therapy (IPT) face to face in the primary care practice. However LHCs faced great difficulties in delivering Interpersonal Therapy (IPT) for two major reasons: first, because patients were unable to attend the required minimum of 6 sessions, and secondly because some patients were skeptical about its potential benefits, preferring medication.

*"Patients told that they won't be able to come for so many sessions. Some patients postponed the appointments and finally they dropped out. Some refused to continue saying they are better and don't need to continue. I felt that IPT is a good therapy but the response in my PHC was poor. Whenever I introduced IPT to patients most of them said that IPT will not help them to deal with their problems; at least with medicines they will feel better." *[LHC]

A few LHCs mentioned that as they gained experience they incorporated some of the IPT components (giving hope, interpersonal inventory, communication and decision analysis) into delivering psychoeducation and that these components were helpful to handle interpersonal problems. These informal actions of the PHC phase were later formalized during the GP phase and extended to all LHCs through a revision of the psychoeducation procedure.

Many LHCs mentioned how the conviction of the primary care staff about the benefits of the program was a critical element in enhancing patient engagement. They reported some challenges with engaging the primary care doctor, especially in the early days when some doctors were not referring patients for counseling or playing an active role in encouraging patients to accept the new treatments. However, the involvement of the doctors increased as they became aware of the positive feedback about the treatment from patients. In addition, some patients who had felt their health improve following counseling also encouraged others to participate and even created awareness in the community about the program.

*"Doctor was convincing patients to meet HC and take benefits of the counseling and to regularly follow up. He was also discussing with me about the treatment of the patients." *[LHC]

#### Supervision by the Clinical Specialist

The role of the Clinical Specialist (CS), a visiting psychiatrist, was to train the doctors in the intervention clinics on the MANAS treatment protocols and use of ADT and to support and supervise the primary care teams in these clinics. Almost all the GPs, but fewer PHC doctors (3/11), mentioned that the CS was supportive and that they had discussed a variety of clinical issues with him, for example, diagnostic difficulties, medication dosage and refractory patients. In the PHCs, several doctors mentioned that they only sought consultation from the CS if facilitated by the LHC who would discuss the case with the CS and then report it to the doctor. Almost all the doctors endorsed the importance of the role of the CS in the program.

"If I had any problem regarding patients, like if the dose had to be increased or patient is having minor side effects like giddiness or gastritis, I definitely consulted him (CS) and asked as to what should be done. The regular follow ups that he made were very useful in knowing the progress of the program. He was always available on the telephone whenever I had any difficulties. It is definitely useful because a psychiatrist is always necessary in running of such programs." [PHC Doctor]

The LHCs also appreciated the CS's support and guidance in dealing with difficult cases, especially patients with high suicide risk. They also mentioned that the CS engagement with the clinic doctors, for example through monthly performance reports to the doctors, played a crucial role in engaging the doctors with the program. The CS observed that there were variations in the degree of enthusiasm and the personal interest by different doctors in the program. CS was able to establish continuing relationships with the GPs with whom training was an ongoing process whereas few PHC doctors were available for supervision due to changing shifts or doctors having other work commitments leading to uneven engagement.

*"There was reluctance to adopt stepped care treatments in spite of repeated reminders during supervisory visits. This was especially a problem with some older doctors who have preconceived notions of depression and found it hard to change them. Arranging for regular supervision with all doctors in the clinic within time and traveling constraints led to uneven support provided to individual doctors." *[CS]

The CS was able to overcome some of these challenges through regular presentation of the program performance to the practice staff, reviewing the program protocol regularly with the doctors, and ensuring regular visits to the clinics and availability on the phone. CS pointed out that there were very few referrals and that these were easily managed by them. CS mentioned that building strategic relationships with key players within the practice was crucial - being on time for meetings, befriending staff in a respectful manner, being non judgmental, enthusiastic, responsive to the needs of the doctors and practice staff, interacting in a non-demanding and supportive manner with them, being persistent and tenacious in sorting out problems, being patient and understanding while encouraging the adoption of the new requirements such as the use of a checklist and changes in reporting format, and highlighting the positive impact of the intervention on patients improved acceptance of the program.

*"The general acceptance of each doctor of the program and their readiness to receive feedback/inputs was of great help. Also, the HC's integrating themselves in the clinic led to greater acceptance of the program. Our availability at all times over the phone was also beneficial." *[CS]

#### Patient adherence

MANAS faced a number of challenges in achieving patients adherence to the program for a variety of reasons; the team generated a range of strategies to overcome these challenges as summarized in Table 2.

**Table 2:** Adherence barriers and strategies adopted

Barriers to adherence	Strategies
**Inability to send reminders: **due to wrong address or wrong phone number; no phone number; not giving consent to send reminder letter.	*Reminders through a variety of modes tailored to each patient, for example: contact through community health workers, phone number of relatives or neighbors.

**Cost: **Medicines, Doctor's fee (GP practices), and travel for sessions.	**Addressing financial difficulties**, for example providing information about widow pension or senior citizen welfare schemes. **Reducing costs**, by prescribing cheaper ADT brands, providing free medication to patients with financial difficulties, long duration prescriptions.

**Unable to come to the clinic: **due to living long distance from clinic; child care responsibilities; unable to get time off work.	**Flexible appointments **according to the convenience of the patient. **Reducing patient waiting time**: registering in advance for doctor's appointment to avoid waiting in queue.

**Age: **Elderly patients forget appointment and also need company to come to clinic.	**Phone Counseling**. **Seeking family support for example**, to collect medication after the phone session with the patient.

**Treatment related: **"felt better" and thus discontinued treatment; side effects of medicine; no improvement with medication; long duration of treatment; no health care answers for life stressors.	**Addressing treatment concerns **for example, joint consultation on side effects with the doctor.

**Lack of engagement of treating doctor and/or clinic staff**.	*Regular feedback to doctor and primary care team about program performance.

**Unavailability of ADT in local pharmacy**.	**Checking and promoting the availability of ADT **in local pharmacy.

Note: * Strategies used in all cases

These included a variety of strategies used by LHCs, sometimes with mixed responses.

"I used to emphasize taking medicines (antidepressants) for at least six months and gave flexible follow-up appointment according to patient's convenience. I am always in my cabin during the appointment timings as some patients were on time ...I remind patients who miss their appointments with phone calls, letters or contacting through PHC field health workers. It is natural that patients can forget their appointment when they are stressed or depressed." [LHC]

*"Many times I am faced with the problem where patients do not like us to send letter to remind them of appointments or phone them. In one instance one patient came and told me "you stop sending letters to me, people are suspicious why letters are coming to me from the clinic. I will continue my medicines". So I stopped but that patient was regular in taking medicine. But there was another patient just opposite; she would come to the clinic only when she receives the reminder letter*." [LHC]

Some doctors reported that some patients complained that there was no improvement with medication, that the treatment was for long duration, or that they didn't come back if they felt better; some patients also felt that there were no health care answers for life stressors.

"When patients feel little better they do not come for treatment. After two months again when the symptoms comes back, they come back again to us. [Doctor]

*"These patients are living through stress and they consider stress as accepted part of life and they feel that what doctor will do about it." *[PHC doctor]

The doctors also provided support for adherence management through information about the treatments and instructing patients to report any discomfort. Longer prescriptions, for example for one month (in PHCs, prescriptions were usually for two weeks duration) were also provided for patients who lived a long distance away. Some GPs did not charge fees for follow-up visits, provided free medicines to a few patients with financial difficulties and prescribed cheaper ADT brands. The LHCs kept up to date lists of available ADT brands in their local pharmacy and their costs to the doctor.

### Practices by control arm general practitioners

In the following sections we describe the practices of the GPs in the control arm, in comparison to those carried out in the MANAS collaborative stepped care approach.

#### Psychosocial interventions

All 6 control arm GPs routinely diagnosed CMD by taking a history which covered both the patient's health and personal and family problems; three explicitly inquired about suicidal behaviours, providing counseling and informing family members. All but one explained the diagnosis to their patients as having a "*psychological problem*". The other GP previously referred to CMD as a "*mental illness*", but switched to using the term "*mental stress*" during the program.

"Those individuals who used to present us with various vague complaints, we used to explain to these types of patients that they don't have any disease and their symptoms are mainly psychological, so you try and take some antidepressants or some anti anxiety tablets, it will help you."

"I would tell them that you don't have any illness and your illness is only of thinking about your family or your home"

All the GPs provided psychosocial guidance for personal or family problems, for example some GPs provided advice on lifestyle change, others gave advice on how patients should distract themselves from their problems and/or share with relatives. They also provided them information on yoga or referral agencies such as Alcoholic Anonymous. Two GPs taught patients problem solving techniques.

"If some patients have family problems then I used to call their relatives or their closest person and explain to them. I also give advice, for example change in the life style, change in the environment in the house whenever it was the cause."

"I used to tell them even earlier also pin point your problems; write down your problems. Once you identify your problems half of it is solved."

#### Medication practice

Prior to the program, four of the 6 GPs routinely prescribed ADT and also gave information about the side effects; the other two GPs who usually prescribed benzodiazepines modified their medication practice to ADT during the program. Half the GPs mentioned that they also prescribed vitamins, and benzodiazepines concurrently with ADT. Half the GPs reported that they would prescribe ADT to patients with mild CMD before the program, but changed to offering counselling for these patients during the program. All the GPs would discontinue the treatment after symptom recovery.

"Once they come back and I know that their problem is getting solved, they are feeling better I start reducing the dose and then gradually I stop the treatment."

#### Specialist referral

Most GPs (4/6) referred patients with severe CMD to a Psychiatrist.

#### Adherence management

Most GPs informed and convinced the patients to continue treatment for longer duration and gave the follow up appointment after every 15 days.

"I tell them (patients) that unless you take these medicines you won't come out of it and to come out of it you have to take the medicines regularly and for longer time. It is not like other medicines for cold and fever where you take the medicines for few days and you are alright. I will give you these medicines, review you and depending on your condition I will increase or reduce the dose."

One GP provided a health record booklet to patients which they needed to bring for every follow up visit, sent birthday greeting cards to patients, and involved family members in adherence management.

At the end of the interview when GPs were informed about the trial finding that patients who were receiving care in their clinic did as well as those who were receiving care in the clinic with a LHC, they explained this finding as being due to patients having "*faith*" in them and the rapport developed with their patients over many years they had been running their practice. They were aware of the patients' family backgrounds, took time to listen to their problems, took detailed information about their problems, and offered treatment and advice accordingly.

*"I think it was because we have good rapport with the patients, they can talk to us better, sometimes before they come to me I know their problems, since childhood I know them and also their families*"

*"I feel that we go into the details of the problems of the people, we talk to them, we come to know their problems and help them. All this helps the patients"*.

## Discussion

The integration of mental health in primary care is one of the most widely established axioms in global mental health; yet, there are few descriptions of the challenges and opportunities in the context of actual interventions aiming to achieve integration. The MANAS trial is the largest evaluation of such an intervention in a developing country. This paper describes the findings of semi structured interviews carried out on completion of the trial, with all the key health care stakeholders involved in both intervention and control arms, in order to explore their experiences of the program. Key innovations of MANAS were its method of case identification by routine screening, the utilisation of a trained Lay Health Counsellor (LHC) as a case manager in the clinics, and the provision of a visiting psychiatrist (the Clinical Specialist) to support the primary care team.

Our main findings are that the primary care doctors found the program to be a valuable addition to primary care, and their acceptance of this program evolved over time as their rapport with and trust of the LHC's competence grew and they observed benefits to patient clinical outcomes. Several components of the intervention were specifically reported to have been valuable: screening and the categorization of the severity of CMD and reporting of the results on a patient card was an aid to the doctor in diagnosis and providing treatment by overcoming the challenges of the shortage of time and the common presentation of somatic symptoms; the LHC provided a range of psychosocial treatments and emphasized adherence management which widened the scope of health care interventions and enhanced the likelihood of success; and the Clinical Specialist supported the team and gave confidence to the doctor to support the program in the clinic, prescribe anti-depressant medication and reduce the use of benzodiazepines and nutritional supplements. The new members of the team (the LHC and Clinical Specialist) also reported that their efforts to integrate the program had greater impact with the passage of time and the observed benefits to patients. Systematic preparation in the form of a comprehensive training program focusing on skills based learning, followed by a structured on-the-job supervision protocol comprising both on-site supervision by the Clinical Specialist and once a month peer-group supervision with other LHCs were cited as critically important elements for successful delivery of the program.

Several challenges were also observed, some of which were resolved during the program but others were not. The hesitance of patients to engage with the intervention reduced in time as the procedures related to the intervention became routine and the clinic staff provided information and reassured patients about the benefits of screening and that they would not miss their place in the queue. Non-adherence was common and was attributed to a number of reasons, often because of the feeling that continuing treatment was not needed as symptoms had been controlled or because of doubt that health care interventions could address one's 'life difficulties'. Interpersonal therapy, provided face to face in the facility, required several visits for hour-long sessions, could not be delivered as planned due to the cost implications related to travel to the clinic and the time lost from work. Cost factors were also a barrier to medication adherence in the GP practices. The LHCs had particular difficulty in working with patients with extreme social difficulties or alcohol related problems, and elderly patients, as the intervention seemed unable to address their specific needs. Some patients dropped out of the intervention because they felt there was no impact on their health or because they were dissatisfied with the length of time taken to help them recover. However, in time the LHCs were accepted by the patients and appreciated by the primary care staff due to their polite and friendly nature and also for maintaining confidentiality. Regular contact by intervention team members with the health facility staff, for example through regular feedback of the progress of the program, helped engage with and enhance the support of the facility staff. The LHCs described a number of strategies they had adopted to improve adherence, tailoring these to address the barriers expressed by specific patients.

Our interviews with the control arm GPs revealed a number of practices which approximated the program interventions to the extent to which these might well explain their comparable performance. Most GPs routinely diagnosed CMD and provided psychoeducation, advice on life style changes and problem solving. Most GPs routinely prescribed ADT and referred patients with severe CMD to a psychiatrist. Above all, GPs developed good rapport with patients, offering one-to-one consultations in a private space, maintaining confidentiality, and offering advice which reflected their long-standing relationship with the patient and understanding of the patient's social context.

In conclusion, our research shows that a trained lay health counsellor is an acceptable addition to the primary health care team for the provision of mental health care and that adequate training, systematic steps to build rapport and trust, the passage of time, the observable impacts on patient outcomes, and the support and supervision by a visiting specialist are key elements to enhance the integration of the mental health care intervention. The primary role of the counsellor is to be a case manager, coordinating continuing care and providing all psychosocial interventions. In the context of the private GPs who participated in the MANAS trial, most of the roles played by the LHC were already being performed by the GP, and thus the control arm GPs were as effective as those with a LHC in enabling recovery. In these practices, the provision of screening for case-detection is a sufficient intervention to achieve comparable outcomes. The effective integration of mental health in primary care, therefore, relies most crucially on a systematic process for the detection of CMD followed by a therapeutic relationship with a primary care provider which addresses the symptoms of CMD through an appropriate combination of psychosocial interventions, antidepressant medication and referral to specialist mental health providers. Future research should focus on the development and evaluation of specific, structured, psychological treatments which address the barriers we experienced with delivering interpersonal therapy, and examine whether the addition of this treatment enhances the effectiveness of the LHC intervention package.

## Abbreviations

ADT: Anti-depressant; CMD: Common Mental Disorders; CS: Clinical Specialist; CSC: Collaborative Stepped Care; EUC: Enhanced Usual Care; GHQ: General Health Questionnaire; GP: General Practitioner; HA: Health Assistant; IPT: Interpersonal Therapy; LHC: Lay Health Counsellor; PHC's: Primary Health Centres.

## Competing interests

The authors declare that they have no competing interests.

## Authors' contributions

All authors have made substantive contributions to the study design or analysis of the data in this paper, have been involved in drafting the manuscript and have approved the final submitted version.
